# Effects of Donkeys Rearing System on Performance Indices, Carcass, and Meat Quality

**DOI:** 10.3390/foods10123119

**Published:** 2021-12-16

**Authors:** Paolo Polidori, Natalina Cammertoni, Giuseppe Santini, Yulia Klimanova, Jing-Jing Zhang, Silvia Vincenzetti

**Affiliations:** 1 School of Pharmacy, University of Camerino, Via Gentile da Varano, 62032 Camerino, Italy; 2 School of Biosciences and Veterinary Medicine, University of Camerino, Via Circonvallazione 93, 62024 Matelica, Italy; natalina.cammertoni@unicam.it (N.C.); giuseppe.santini@unicam.it (G.S.); yulia.klimanova@unicam.it (Y.K.); jingjing.zhang@unicam.it (J.-J.Z.); silvia.vincenzetti@unicam.it (S.V.)

**Keywords:** donkey, carcass quality, meat quality, pasture, fatty acids profile

## Abstract

This study compared growth rates, carcass, and meat quality obtained from 24 male crossbred donkey foals reared for meat production under an intensive (I) or extensive (E) feeding system. Donkeys were slaughtered at 16 months of age; the average final body weight, hot and cold carcass weight, and hot and cold dressing percentage were significantly higher (*p* < 0.05) in the I group. Samples of *Longissimus Thoracis et Lumborum* (LTL) were taken from each foal for chemical and physical analysis. Group I showed significant (*p* < 0.05) higher intramuscular fat, while the E group showed significantly (*p* < 0.05) higher protein and unsaturated fatty acids (UFA) contents, including n-3 essential fatty acids. Saturated fatty acids (SFA) and glycogen were significantly higher (*p* < 0.05) in the intensive system, monounsaturated fatty acids (MUFA) were significantly higher in the extensive system. The ratio PUFA/SFA was significantly higher (*p* < 0.05) in group E. The Thrombogenic Index (TI) was significantly higher (*p* < 0.05) in the I group. Meat tenderness was significantly higher (*p* < 0.05) in group I. The feeding system had no effect on cholesterol content and meat color characteristics. Donkeys average daily gain, carcass weight, and some meat quality parameters were significantly affected by the rearing system.

## 1. Introduction

The development of modern farming techniques, including the increase of motorized vehicles, caused a marked decrease in the use of donkeys as beasts of burden, basically employed in the transportation of goods and people [1,2]. About 44 million donkeys are registered worldwide (Table 1). More than 60% are reared in Africa and Asia. There are 189 donkey breeds worldwide (more than 50 breeds in Europe [3,4]), showing different body weights (from 80 to 480 kg). Among the smallest breeds are the Mediterranean Miniature Donkey, and among the largest is the American Mammoth Jackstock.

Donkey meat has never been recognized as a high-quality food, mainly because of the toughness associated with this product [5]. China has the largest donkey meat production, followed by Niger (Table 2). Among equid meat, a different reputation is attributed to horse meat, which is generally considered a good alternative to red meat, with healthy properties appreciated by the consumers. For these reasons, in Spain, France, and Poland, a significant increase in horse meat consumption has been reported in recent years [6,7]. Equid meat is characterized in both horse and donkey by low total fat and cholesterol content, a positive ratio between saturated and unsaturated fatty acids, and good iron content [8]. Consumers today are very interested in leaner meat with low fat content, and also pay great attention to animal feeding management [9]. The extensive feeding systems, largely used in donkey breeding, are preferred by a significant number of consumers because feeding the animals on natural pastures can have a great effect on meat and carcass quality, as previously reported on Navarra and Burguete foals [10,11], while scarce data are available about the effects of different feeding strategies on donkey meat.

Donkey carcass yield is affected by four main parameters: animal age, genotype, gender, and livestock production system [12]. Horse carcass quality traits were not significantly affected by production systems and feeding strategies [13]. Consumer’s interest in donkey meat is influenced by the perception of a food’s “healthiness” that is mainly related to intramuscular fat content, fatty acid profile, and cholesterol content [14]. This study was performed to analyse and compare the effects on performance indices, carcass yield, and meat quality obtained from 24 male entire male crossbred donkey foals reared under an extensive and intensive production system and slaughtered at 16 months of age.

## 2. Materials and Methods

### 2.1. Animals and Diets

This study was approved after its completion by the Animal Welfare Committee of the University of Camerino (Ref. July 2021). Twenty-four crossbred (Martina Franca x Ragusana) entirely male donkey foals reared in the same farm were used in this study. Foals naturally suckled colostrum at birth and later milk from their dams till the weaning at the age of seven months; starting from 3 months of age, they also received oat hay *ad libitum*. After weaning at the age of seven months, donkeys with similar average body weight (128 ± 9.2 kg) were selected and randomly assigned to two experimental groups. The study started in autumn 2019 (October) and lasted till June 2020, just prior to the beginning of the summer season. It was performed on a farm named “Mamma Asina”, located in Colmurano, Macerata Province, Le Marches Region, Italy. Twelve donkeys were reared under the intensive system with *ad libitum* access to natural grass hay and 400 g/day/head of commercial concentrate feed supplied twice per day (6:30 a.m. and 7:30 p.m.). The ingredients of the commercial concentrate feed were, in unknown quantities, barley, soy meal, dry beet pulp, beet molasses, wheat germ, calcium carbonate, sunflower oil, sodium chloride, and a vitamin and mineral mix. The other twelve foals, reared under the extensive system, went out to graze *ad libitum* from morning until dark during the nine month experimental period. The extensive feeding system was based on natural pastures improved by sowing perennial ryegrass (*Lolium perenne*); the chemical composition of all feeds used in this study are shown in Table 3. All the 24 donkeys in the stable had free access to water.

### 2.2. Slaughter Procedure, Carcass Evaluation, Sampling, Chemical and Physical Analysis

At around 16 months of age, foals were slaughtered following the standard practices mentioned in the EU rule n. 1099/2009. Donkeys fasted for 12 h before slaughter, and only water was available. Before slaughtering the donkeys, final body weight had been detected, and average daily weight gain was calculated. All the animals were weighed 4 times: on the 1st day of the trial (October 2019), on 9 January 2020, on 18 April 2020, with the final weighing coming on the 24th just before slaughter. Average daily weight gain was calculated considering the difference between the average final body weight registered before slaughter on day 240th and the initial average body weight (128 ± 9.2 kg) at the beginning of the study, divided for the length of the study (240 days).

Carcasses were skinned and eviscerated; 1 h after slaughter, hot carcass weight and hot carcass dressing percentage were calculated. In the following 24 h, carcasses were stored in a cold room at 2 °C, then cold carcass weight and cold carcass dressing percentages were determined. The dressing percentage was calculated using the formula:

Hot Dressing % = (hot carcass weight/final body weight × 100

Cold Dressing % = (cold carcass weight/final body weight) × 100

From the left side of each cold carcass, samples of *Longissimus Thoracis et Lumborum* (LTL) muscles were removed 24 h after slaughter between the 13th and 16th vertebra (about 400 g of each sample) and transported in refrigerator bags (4 °C) to the lab, where chemical composition and cholesterol content were determined in all the samples [15]. Residual glycogen was determined spectrophotometrically (340 nm) [16]. Fatty acid composition was determined after lipids extraction [17], and later fatty acid methyl esters were used for determining the fatty acid profile [18]. A gas chromatograph (model CP 9003, Agilent Technologies, Santa Clara, CA, USA) with a flame ionization detector and a fused-silica capillary column and a film thickness of 0.2 μm, packed with CP Sil 88 (50 m × 0.25 mm i.d.), was used. Fatty acid identification was performed by comparing the antioxidant standard butylated hydroxytoluene (BHT) with gas chromatographic retention times. Atherogenic index (AI) and thrombogenic index (TI) were calculated using the equations:

AI: (C12:0 + 4 × C14:0 + C16:0)/Mono Unsaturated Fatty Acids (MUFA) + Poly Unsaturated Fatty Acids (PUFA n-6 + PUFA n-3)

TI: (C14:0 + C16:0 + C18:0)/(0.5 × MUFA) + (0.5 × PUFA n-6) + (3 × PUFA n-3) + (PUFA n-3/PUFA n-6)

to provide specific lipid quality parameters, indicating that C12:0, C14:0, and C16:0 are atherogenic and that C14:0, C16:0, and C18:0 are thrombogenic [19].

Shear force determination was determined on LTL muscles seven days *post mortem*: chops (each 2.5 cm thick) were roasted at an oven temperature of 75 °C, an internal temperature of 70 °C was monitored with thermocouples [3]. From each sample, 8 cores (1.2 cm in diameter) were sheared with a Warner-Bratzler operating head mounted on an Instron apparatus 4411 (Instron, High Wycombe, UK). Peak or maximum shear force was expressed in N/cm^2^.

Muscle color parameters were measured 48 h after slaughter after 1 h of oxygen exposure at room temperature using a Minolta CM-3600 D spectrophotometer (Konica Minolta Holdings Inc., Marunouchi, Chiyoda, Tokyo, Japan), with a measured area diameter of 8 mm, a standard illuminant D65, and an observer angle of 10°. White calibration was made with the standard tiles (Y = 85.3, x = 0.3173, y = 0.3251) in order to determine the L* (lightness), a* (redness), and b* (yellowness) [20] on a freshly cut surface after 30 min of blooming at room temperature (21 °C). After placing the measuring lens on the meat surface, it was turned through 0°, 45°, and 90° (clockwise) to obtain three different reflectance measurements that were later averaged.

### 2.3. Statistical Analysis

The statistical significance of differences between production systems was determined by the Student’s *t*-test using the SAS software [21]. Each parameter was tested for normal distribution and normalized. Each animal was considered an experimental unit and used as a random variable. All the data were expressed as least-square means; the variability of the data is shown by standard mean error (s.e.); statistical significance was set as *p* < 0.05.

## 3. Results

Table 4 shows results concerning the donkeys’ final body weight, average daily gain, and carcass quality parameters. Animals belonging to the intensive system showed a significant (*p* < 0.05) higher final body weight (252.1 ± 25.2 kg) compared to the extensive system group (238.5 ± 21.2 kg). Growth rates registered in this study were significantly higher (*p* < 0.01) for the intensive system; the foal’s growth obtained in this study was similar to the results determined on donkey foals slaughtered at 12 and 18 months of age [22]. In the intensive system, both hot (139.1 ± 11.4 kg) and cold carcass weight (136.9 ± 6.23 kg) were significantly (*p* < 0.05) higher compared to the results registered in the extensive system (126.6 ± 10.1 kg and 122.3 ± 8.34 kg, respectively).

Farm management significantly affected donkey meat chemical composition (Table 5). The extensive system provided donkey meat with a significantly higher (*p* < 0.05) protein content (21.7%) compared to the intensive (20.4%).

On the other hand, intensively-reared donkeys showed a significantly (*p* < 0.05) higher amount of intramuscular fat (IMF) compared to the extensive system (1.35 vs. 1.10%, respectively). The higher IMF content in the intensive system could be associated with the reduced physical activity of donkeys reared under intensive production systems [23]. Glycogen content was significantly (*p* < 0.05) lower in the extensive system (0.32%) compared to the intensive system (0.46%), while cholesterol content was not affected by feeding management.

No significant differences were observed between the color parameters lightness (L*), red (a*), and yellow (b*) in meat obtained from both groups (Table 5). Similar trials in which quality parameters of meat produced by lambs [24] and beef cattle [25] have been investigated associated the darker meat (lower L*) obtained from animals reared under extensive breeding systems with the higher myoglobin content detected in their muscles. This parameter has not been investigated in the present study; the higher IMF determined in the intensive system can be associated with an increase of the L* value of the meat produced in this group of animals, due to the high light reflection/scattering property of fat [26].

Meat tenderness was affected by the feeding system (Table 5). Donkeys belonging to the intensive system showed significant lower (*p* < 0.05) shear force values seven days after slaughter (49.4 N) compared to the extensive system (58.5 N), confirming the effect of livestock production systems on meat tenderness in foals slaughtered at 18 months of age [27].

The donkey meat lipid profile is shown in Table 6. The two most represented fatty acids were oleic acid (C18:1cis9) and palmitic acid (C16:0) in both groups, confirming the results obtained in horse [28] and donkey meat [29]. Meat produced by animals reared under the extensive feeding system showed a significantly (*p* < 0.05) higher content of polyunsaturated fatty acids (PUFA) compared to animals reared under the intensive system, 28.08 and 26.20 g/100 g total fatty acids (Table 6), respectively. Saturated fatty acids (SFA) content was significantly higher (*p* < 0.05) in the intensive system (42.07 ± 1.99) compared to the extensive system (38.51 ± 2.05), while monounsaturated fatty acids (MUFA) content was significantly higher in the E group (33.42 vs. 31.73, respectively). The ratio between PUFA/SFA was significantly higher (*p* < 0.05) in the extensive system (0.73 vs. 0.62); both these values are close to the upper limits (≥0.45–0.7) recommended by the health authorities [30]. Considering the PUFA n-6 and n-3 series, linoleic acid (C18:2n-6) represents the most abundant among the n-6 family, as determined in previous studies [31]. Within PUFA n-3, α-linolenic acid (C18:3n-3) content is normally lower than 30% of total PUFA, even if higher values have been found in meat from horses either extensively reared or receiving low concentrate feeds [32]. The total amount of n-3 PUFA determined in this study was significantly higher (*p* < 0.01) in the extensive system (5.50 vs. 3.36). EPA content was significantly (*p* < 0.01) higher in the extensive system (0.20 ± 0.03 g/100 g total fatty acids) compared to the intensive system; docosahexaenoic acid (DHA; C22:6n-3) was not detected. Meat from the extensive system showed a significantly (*p* < 0.01) lower ratio (4.10) among n-6 and n-3 PUFA compared to that one (6.80) determined in the intensive system. AI was not significantly different among the two groups of animals, while TI was significantly higher (*p* < 0.05) in the I group.

## 4. Discussion

In the present study, the foal growth rate was significantly higher in donkeys reared using the intensive system compared to donkey foals reared in the extensive system, confirming the results provided in a study in which an intensive farming system based on linseed feed supplementation produced foals with significantly higher carcass weight and dressing percentage [33]. These results must be considered preliminary data which must be integrated into future studies, in which grass intake during donkeys extensive feeding in a natural pasture can be estimated, and more accurate results regarding feed conversion rates can be obtained.

Regarding donkey meat chemical composition, this study confirmed that protein content in donkey meat is similar to other red meats [34]; because of their low growth rate, donkeys normally convert energy first into bones and muscles and later into fat [35]. In fact, in older donkeys it is possible to determine a higher amount of IMF compared to younger animals [36]. Results obtained in this study confirmed that animal diet can significantly affect IMF and its fatty acid profile [37]. In equid meat, it is possible to determine small amounts of residual glycogen, even if significant differences can be detected among different muscles [38]. The results obtained in this study confirmed that feeding management does not affect cholesterol content, as previously determined on other animals reared for meat production [39].

Meat tenderness results determined in this trial were very close to those obtained in muscle LTL taken from Martina Franca foals slaughtered at 18 months of age [14], but they were markedly higher when compared to the shear force values determined on muscle LTL taken from 12 month old Italian Heavy Draft male horses [40]. Taking into account the actual reputation of “tough meat” that is often associated with donkey meat [41], improving donkey meat tenderness with adequate aging periods and appropriate animal diet is a crucial strategy to supply tender meat and acquire new market share.

Slaughter age significantly affects α-linolenic acid n-3 derivatives contents in horse meat, with higher content of C18:3n-3 in younger foals, while C20:5n-3 (eicosapentaenoic acid, EPA) resulted in greater amounts in older foals [7]. Both linoleic and α-linolenic acid are essential fatty acids, because they cannot be synthesized by the human body and must be introduced into human diets [42]. The n-6/n-3 fatty acids ratio represents an important food quality parameter that should remain under 4 because the decrease of n-3 PUFA consumption in the human diet can create the right conditions for developing severe diseases, such as cancer and cardiovascular diseases [14].

Among trans FAs, reported in horse meat as 9t- and 11t-C18:1, it is known that ruminants’ meat has markedly greater CLA (conjugated linoleic acid) concentration compared to meat produced by monogastric animals [43]. For this reason, equid meat shows only trace CLA content compared to beef or lamb [44], as this study also confirms. The higher value of TI determined in the intensive system indicates potential risk to human health, as also determined in donkey meat derived products [45].

In this study, donkey meat fatty acids are predominated by SFA, as also determined in meat obtained by horses fed with concentrate [46]. Other studies showed PUFA as predominant in meat lipid profiles from foals of different breeds reared under a semi-extensive system, or in animals managed under extensive livestock systems [47].

Animal diet confirmed its crucial role on meat fatty acids composition; the extensive system significantly improved the fatty acid profile of meat, significantly reducing SFA and significantly increasing PUFA contents. Donkey meat can represent a potentially good quality alternative for human consumption among red meats considering its low-fat content and the healthy fatty acid profile of meat obtained under an extensive system.

## 5. Conclusions

The production system (intensive and extensive) significantly affected donkey carcass traits and meat chemical composition. Feeding donkeys using the extensive system produced lighter carcasses, while donkey meat showed lower values of IMF and higher levels of EPA, n-3 fatty acids, and a higher PUFA/SFA ratio, providing appealing quality characteristics for health-conscious consumers. Tenderness was significantly affected by the production system, showing lower shear force values in meat produced under the extensive system. This study supplies new information that can be used to increase donkey meat production and consumption, providing new targets for research on the usage of donkeys as meat-producing animals. Further studies regarding the evaluation of the best slaughtering age and the effects of appropriate feeding strategies on donkey meat are necessary.

## Figures and Tables

**Table 1:** World Donkeys Distribution (2018).

Continent	Donkeys (*n*)
Asia	15,000,000
Africa	9,700,000
Middle East	9,220,000
South America, Caribbean	8,164,000
Europe	1,500,000
North America	52,000
TOTAL	43,636.000

Source: [5].

**Table 2:** Donkey meat production in the world (2018).

Country	Donkeys (*n*)	Meat Production (Tons/Year)
China	2,249,807	183,755
Niger	124,319	9946
Burkina Faso	72,372	4342
Senegal	52,581	3155
Mali	50,598	3036
Mauritania	22,920	2521
World	2,569,520	207,172

Source: [5].

**Table 3:** Chemical composition of experimental feeds (g/100 g Dry Matter).

Feed	Natural Grass Hay	Natural Pasture	Concentrate Feed
Dry Matter %	91.58	44.86	94.00
Ash	6.11	11.29	4.58
Crude Protein	4.36	10.6	16.0
Ether Extracts	1.20	0.58	1.50
NDF	65.7	63.56	36.61
ADF	39.0	34.93	8.34
ADL	4.85	4.91	1.81

NDF: Neutral Detergent Fibre; ADF: Acid Detergent Fibre; ADL: Neutral Detergent Lignin.

**Table 4:** Donkey Body Weight and Carcass Parameters (mean ± s.e.).

Quality Trait	Intensive System (*n* = 12)	Extensive System (*n* = 12)
Final body weight (kg)	252.1 ± 25.2 ^a^	238.5 ± 21.2 ^b^
Average daily gain (g/d)	52.2 ± 18.4 ^B^	46.8 ± 14.7 ^A^
Hot carcass weight (kg)	139.1 ± 11.4 ^a^	126.6 ± 10.1 ^b^
Hot dressing %	55.2 ± 0.94 ^a^	53.1 ± 0.90 ^b^
Cold carcass weight (kg)	136.9 ± 6.23 ^a^	122.3 ± 8.34 ^b^
Cold dressing %	54.3 ± 0.85 ^a^	51.3 ± 0.81 ^b^

Different letters in the same row indicate a significant difference (a, b: *p* < 0.05; A, B: *p* < 0.01).

**Table 5:** Chemical and Physical Parameters of Donkey Meat (mean ± s.e.).

Chemical Composition	Intensive System (*n* = 12)	Extensive System (*n* = 12)
Moisture %	76.5 ± 2.88	75.6 ± 3.21
Protein %	20.4 ± 1.76 ^a^	21.7 ± 1.26 ^b^
Intramuscular fat %	1.35 ± 0.25 ^a^	1.10 ± 0.32 ^b^
Ash %	1.29 ± 0.14	1.28 ± 0.47
Glycogen %	0.46 ± 0.08 ^a^	0.32 ± 0.06 ^b^
Cholesterol (mg/100 g)	66.7 ± 3.53	67.3 ± 3.35
**Color parameters (48 h)**		
L*—lightness	35.7 ± 0.37	34.4 ± 0.40
a*—redness	11.4 ± 0.19	12.1 ± 0.23
b*—yellowness	7.7 ± 0.12	7.6 ± 0.13
Shear force 7 d (N/cm^2^)	49.4 ± 2.15 ^b^	58.5 ± 2.48 ^a^

Different letters in the same row indicate a significant difference (a, b: *p* < 0.05).

**Table 6:** Fatty acids composition (% total fatty acids) determined in LTL muscles of donkeys reared under the extensive or intensive breeding system (mean ± s.e.).

Fatty Acid	Intensive System	Extensive System
C12:0	0.27 ± 0.01	0.20 ± 0.03
C14:0	3.87 ± 0.53	3.25 ± 0.47
C15:0	0.46 ± 0.14	0.38 ± 0.11
C16:0	27.4 ± 2.46	28.9 ± 2.85
C16:1	3.62 ± 0.31	3.99 ± 0.28
C17:0	0.59 ± 0.17	0.48 ± 0.15
C18:0	7.98 ± 0.99	6.80 ± 1.01
C18:1n-9	27.8 ± 3.11	29.1 ± 3.25
C18:2n-6	20.2 ± 2.22	20.1 ± 2.14
C18:3n-3	3.18 ± 0.49 ^a^	5.20 ± 0.48 ^b^
C20:1n-9	0.31 ± 0.06	0.33 ± 0.05
C20:2n-6	0.19 ± 0.03	0.20 ± 0.02
C20:3n-6	0.17 ± 0.01	0.17 ± 0.01
C20:4n-6	2.28 ± 0.22	2.11 ± 0.24
C20:5n-3	0.18 ± 0.05 ^a^	0.30 ± 0.03 ^b^
SFA	42.07 ± 1.99 ^a^	38.51 ± 2.05 ^b^
MUFA	31.73 ± 1.66 ^a^	33.42 ± 1.18 ^b^
PUFA	26.20 ± 2.00 ^a^	28.08 ± 1.85 ^b^
PUFA/SFA	0.62 ± 0.09 ^a^	0.73 ± 0.12 ^b^
Σn-3	3.36 ± 0.28 ^A^	5.50 ± 0.39 ^B^
Σn-6	22.84 ± 2.19	22.58 ± 1.74
Σn-6/Σn-3	6.80 ± 0.55 ^B^	4.10 ± 0.56 ^A^
Atherogenic Index—AI	0.57 ± 0.03	0.50 ± 0.04
Thrombogenic Index—TI	1.09 ± 0.31 ^b^	0.84 ± 0.12 ^a^

Different letters in the same row indicate a significant difference (a, b: *p* < 0.05; A, B: *p* < 0.01).
